# Differential cannabinergic effects on temporal perception and production

**DOI:** 10.1038/s41386-025-02262-5

**Published:** 2025-10-13

**Authors:** Mario G. Martínez-Montalvo, Diana I. Ortega-Romero, Ana S. Báez-Cordero, Oswaldo Sánchez-Lobato, Claudia I. Pérez-Díaz, Pavel E. Rueda-Orozco

**Affiliations:** 1 Departamento de Neurobiología del Desarrollo y Neurofisiología, Instituto de Neurobiología, UNAM, Campus Juriquilla, Querétaro, México; 2 Posgrado en Ciencias Biológicas. Universidad Nacional Autónoma de México (UNAM), México, México

**Keywords:** Sensorimotor processing, Learning and memory

## Abstract

Cannabinoids have traditionally been associated with motor and cognitive impairments, including slowness of movement and altered temporal perception. However, it remains unclear whether cannabinoids specifically affect the perception and/or production of temporal intervals. To explore these possibilities, we evaluated the effects of systemic administrations of the synthetic cannabinoid CP55940 on behavioral performance in male rats trained in three distinct paradigms designed to assess time interval perception and production. Systemic administration of CP55940 caused temporal overestimation in a fixed-interval task, which was primarily linked to impaired perception of elapsed time in the range of tens of seconds. In contrast, while the same treatment increased forelimb reach duration in a two-interval production task (in the hundreds of milliseconds range), these effects were more accurately attributed to a general reduction in movement speed rather than altered temporal processing. These findings were further confirmed in a third motor task, where animals executed a complex timed motor sequence with spatiotemporal constraints while running on a treadmill. Here, CP55940 administration slowed locomotion but did not disrupt motor timing. Our results demonstrate that, in addition to inducing motor slowness, systemic cannabinoid administration impairs temporal perception but preserves interval production, suggesting distinct underlying mechanisms for these two processes.

## Introduction

Numerous studies have reported that cannabinoid administration alters movement control and time perception. While there seems to be a consensus that low concentrations of cannabinoid agonists induce hyperlocomotion and high concentrations lead to hypomobility [1–3], it is not entirely clear how the cannabinoid system affects time perception [4–6]. Preclinical studies on humans often report that cannabis users experience time as running slowly [7, 8]. This distortion results in an overestimation of elapsed time; that is, subjects report that more time has passed than actually has. On the other hand, behavioral protocols where subjects are required to produce a time interval often report underestimations (i.e., subjects produce longer intervals than required[7, 9]); Studies have obtained similar results in rodents, demonstrating that systemic administrations of cannabinoids also induce an overestimation of elapsed time in “peak interval” procedures [6, 10] or in interval discrimination procedures [5]. However, because time is not specifically sensed by a particular system or region of the brain [11], it has been proposed that the passage of time is sensed by a “distributed clock,” a network of structures including the motor and premotor cortices, the thalamus, the basal ganglia, the hippocampus, and the cerebellum [12, 13]. These regions are linked to both motor and non-motor functions, and it has been proposed that various behavioral processes are involved in time interval estimation, differentiating between behaviors that involve estimating intervals associated with movement execution (interval and pattern motor timing) and those related to sensory stimuli (interval and pattern sensory timing) [14, 15]. However, it remains unclear whether these processes or behaviors share the same anatomical, neurophysiological, and pharmacological bases. In this context, cannabinoid receptor 1 (CBr1) is highly expressed in most of the brain regions typically associated with temporal processing, including the cerebellum, basal ganglia, and hippocampus, which show the highest levels of CBr1 [16, 17]. This anatomical distribution suggests not only that various brain regions are implicated in temporal processing, but also that cannabinoids may affect aspects of temporal processing depending on their specific expression levels or localization within a given structure. On the other hand, certain time-processing effects attributed to cannabinoids may be more related to motor effects that influence timing; for instance, if an animal is slow, it will take more time to complete a given task. Hence, the covariance between temporal and motor parameters during execution in behavioral protocols may compromise interpretations on the exact nature of temporal processing, a topic that has recently been experimentally and theoretically studied elsewhere [18–20]. In this study, we addressed the possibility that covariance in temporal and execution parameters may mask the true nature of the behavioral effects of systemic administrations of synthetic cannabinoids in rodents. To this aim, we implemented three behavioral protocols to simultaneously evaluate movement kinematics (speed/amplitude), as well as motor and sensory timing across multiple time scales, while examining cannabinergic modulation.

## Materials And Methods

Experiments were approved by the Animal Ethics Committee of the Institute of Neurobiology (protocol 102.A) and followed NIH guidelines. All efforts were made to minimize suffering and reduce animal use.

### Animals

A total of 29 Long-Evans male rats (300–650 g) were used and housed under controlled conditions (23 °C, 66% humidity, 12/12 light-dark cycle; all experiments conducted during the light phase) with ad libitum food/water. Seven rats were trained in the fixed-interval task and eight rats in the two-interval production task under water access restricted to training sessions (20–30 mL). Weight was maintained over 85% of the expected weight for their age, with supplemental water if needed. Training occurred 6 days/week, with free access to water on the 7th. Fourteen additional rats performed the spatiotemporal task without water restriction (60–130 trials/day, 6 days/week).

### Behavioral apparatus and training

#### Fixed-interval schedule

**Fig. 1:**
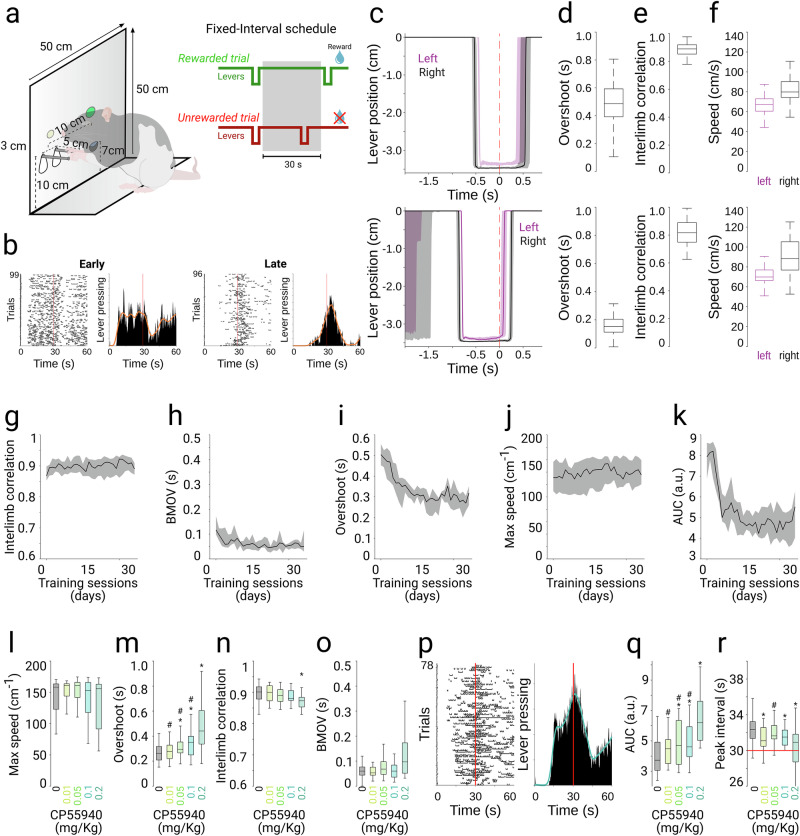
CP55940 effects on the perception and production of temporal intervals. **a** Schematic representation of the behavioral set-up and task structure. **b** Representative lever press rasters (left panels) and peri-reward histograms (right panels) during an early and a late training session. **c** Representative average (shaded area 25^th^ and 75^th^ percentiles) trajectories the left and right levers (color coded) aligned to the reward onset (dotted red line) during an early (up) and late (bottom) training session. Boxplot representation of overshoot (**d**), interlimb correlation (**e**), and speed (**f**) for the same early (upper panels) and late (lower panels) sessions depicted in C. Average learning curves for a group of animals (*n* = 7) for the following variables: Interlimb correlation (**g**), bilateral movement onset variability (BMOV, **h**), overshoot (**i**), maximum lever speed (**j**) and area under the curve (**k**). Data for learning curves are presented as median (solid line) + 75th and 25th percentiles (shaded area). Boxplot comparison of the effect of the CP55940 doses (color coded) on the bilateral execution variables (**l–o**). **p** Representative lever press raster and peri-reward histogram during one session under the highest dose of CP55940. Box plot comparison of CP55940 effects on the peri-reward histogram area under the curve (**q**) and moment of the peak lever response around reward onset (**r**, peak interval). Boxplots represent median and 25th and 75th percentiles. * and # represents significant differences (LSD post hoc test, *p* < 0.05) against control (0 mg/kg) and 0.2 mg/kg conditions, respectively.

Rats were trained in custom-made behavioral plexiglass boxes (50 × 50 x 50 cm) [21–23] equipped with two sets of levers (only the left set was used), a water port, a green LED to indicate correct trials, and a white LED to indicate the availability of the set of levers (Fig. 1a, left). Each set of levers consists of two levers protruding 5 cm from the wall. The levers moved vertically, were connected to a voltage transducer (3.5 cm = 2.5 V), and were continuously digitized and stored at 250 Hz through National Instruments cards (NI PXIe-6363) and LabView custom-made routines. Animals were rewarded with water delivered through the central water port using a solenoid valve. Rats were acclimated to training conditions (40 min) before water restriction began. Initial sessions taught them to obtain water rewards by approaching the port and subsequently by lever touches ( > 0.1 cm displacement). Then, the spatial threshold was progressively increased to 2.6 cm, which all animals learned within two sessions. Then, animals learned to displace both levers simultaneously, and a temporal threshold was introduced (starting at 50 ms, increased to 750 ms over 3–5 sessions). Rats were trained with these parameters for 100–120 sessions before transitioning to a fixed-interval schedule. Sessions lasted 40–60 min with unlimited trials (typically 60–90 trials/session). All execution parameters were based on the raw position data from the levers.

#### Two-interval production task

Animals were trained in the same behavioral chambers and following the same procedure as in the previous task excluding the fixed-interval component. After session 120, the two-interval production schedule was introduced and structured as alternating 20 trials blocks, requiring rats to maintain the levers pressed for 1250 ms during block one (indicated by a blue LED) or 750 ms during block two (indicated by a white LED). After an inter-trial interval of 1.5 s, the levers were available so that the animals could freely perform the next trial. Most of the animals completed more than 200 trials per session, but because they were water-deprived, their engagement in the task was higher at the beginning of the session, performing more than half of the trials in the first 10–12 min. Hence, our analyses were conducted in the first 100 trials of each session. After trial 120, the “block” schedule was suspended, and all trials required the 1250 ms temporal threshold. Pharmacological manipulations were performed after at least 50 sessions of training in the two-interval production schedule. Both protocols, the fixed-interval and the two-interval production task, involved continuous monitoring of efficiency, movement coordination, and execution parameters.

##### Efficiency parameters

To assess task engagement, we calculated the amount of time taken to reach the first 100 trials in each session and the total number of trials per session. For each trial, we measured the “effort” (accumulated duration of lever presses) and the number of attempts required to obtain a reward. A highly efficient trial would involve a single attempt lasting approximately 750 ms (or 1250 ms), plus a few milliseconds of overshoot. Longer movements slightly increased effort but increased the probability of reward (i.e., fewer attempts). Conversely, shorter movements reduced reward probability, increasing both the number of attempts and the accumulated effort.

##### Bilateral movement coordination parameters

To assess bilateral accuracy, we evaluated “interlimb correlation”, defined as the Pearson correlation coefficient between the position of the left and right levers from the beginning of the successful bilateral movement to the delivery of the reward. We also assessed bilateral movement onset variability (BMOV), defined as the variance of the absolute difference of the onset time between each forepaw movement’s initiation expressed in seconds.

##### Execution parameters

To evaluate movement duration, we measured movement “overshoot,” defined as the time the levers remained pressed after the reward was given. “Overshoot fraction” was defined as the magnitude of the overshoot relative to the required interval. For example, an overshoot of 150 ms corresponds to a fraction of 0.2 for the 750 ms interval but 0.12 for the 1250 ms interval. Overshoot and overshoot fraction reflect a motor plan (i.e., programmed movement duration) but also provide insight into whether animals were simply waiting for the reward (i.e., reaction time). Previous studies in rodents reported reaction times of about 300 ms [24, 25]. Hence, overshoots exceeding 300 ms may reflect reaction times to reward delivery, while shorter overshoots likely reflect a preplanned movement duration. We also assessed forelimb movement speed (instantaneous difference in position in 4 ms time bins) and the variability of individual forelimb trajectories. Execution parameters are not necessarily linked to bilateral coordination and likely reflect general features of the motor plan, and unlike bilateral coordination parameters, execution parameters are sensitive to unilateral striatal lesions [22].

#### Spatiotemporal task

The apparatus (modified human treadmill, NordicTrack T6.1) was equipped with Plexiglas walls (50 cm high) to restrict rats to the passable area of the belt (80 cm long by 20 cm wide). The treadmill motor was controlled by a custom-made program (LabVIEW, National Instruments) and a multifunction computer board (NI USB-6353, National Instruments). A line of LEDs illuminated the apparatus, and the front wall was equipped with a drinking spout to deliver drops of sucrose solution. A laser-based photodetector gate (10 cm from the front wall) delimited the goal area. Rats were trained to perform a stereotyped sequence of movements in at least 7 s while running on a motorized treadmill at different speeds. Animals were habituated to the setup, progressively adapting treadmill speeds increasing from 5 to 30 cm/s over 3 90 min sessions (one per day). Training then continued at a fixed speed (30 cm/s, 60–130 trials per session). Correct trials, (entering the goal area after 7 s) were rewarded with a green light and sucrose. Incorrect trials (early entrances) triggered a 1.5 kHz, 65 dB auditory tone lasting 20 s. During each session, the performance average was continuously monitored over the last 40 trials. Sessions ended upon meeting one of three criteria: 1) over 72.5% of correct trials at any point; 2) 60 correct trials; 3) 130 total trials. Once animals met criterion 1 for ≥3 consecutive sessions, we implemented a random speed protocol. Throughout the training, animals were continuously recorded with a high-speed CCD camera (acA640-120fc, Basler, 100 frames/s, 9 pixels/cm) positioned laterally to the treadmill. The animals’ positions were automatically extracted with a custom-made program (Vision, National Instruments) by calculating the center of mass. To quantify behavioral stereotypy, we extracted the position and speed time-courses from each trial across all sessions. Position or speed trajectories were aligned to the entrance times (i.e., end of the movement sequence).

### Administration of the cannabinergic agonist

Systemic administration of the synthetic cannabinoid agonist CP55940 (Sigma and Cayman) was performed with vehicle, 5% dimethyl sulfoxide (DMSO; Merk) + 5% Cremophor (Sigma) in saline. Drugs were injected in volumes of 1 ml/kg. For all tasks, CP55940 concentrations of 0.01, 0.05, 0.1, and 0.2 mg/kg were injected in individual sessions with at least two drug-free sessions between CP55940 injections. All injections were performed 15 min before the behavioral sessions. All animals received at least two injections of each dose. This administration schedule was selected based on previous reports indicating that systemic administrations of the same compound and comparable doses significantly decreased speed and altered prefrontal, hippocampal, and basal ganglia activity within 5–10 min after the injections, effects that persisted for at least 2 h [21, 26–28]. Since each animal received at least two intraperitoneal (i.p.) injections per cannabinoid dose, and the lowest dose (0.01 mg/kg) showed no statistically significant behavioral effects, we opted to forgo additional vehicle injections.

### Statistical analysis

Behavioral data are presented as a median +25^th^ and 75^th^ percentiles. Normality was tested with the Kolmogorov-Smirnov test. Statistical comparisons between groups were performed with the Mann-Whitney test, as stated in the corresponding section. For the effects of multiple doses of cannabinoids over single variables, we applied the Kruskal-Wallis (K-W) test. For the two-interval task, two factors were used: cannabinergic dose and the produced interval. In that case, we applied the two-way extension of the Kruskal-Wallis test, the Scheirer-Ray-Hare (S-R-H) test [29]. An LSD post hoc test was used for multiple comparisons. Statistical analysis was performed using MATLAB software (The MathWorks, Inc.). Statistical differences were considered significant if *P* values were < 0.05.

## Results

### Systemic administrations of CP55940 differentially impact in the perception and production of temporal intervals

We implemented a modified version of a bimanual coordination behavioral protocol for rats [21–23]. Animals were trained to vertically displace two levers simultaneously by a minimum of 2.6 cm and for a duration of at least 750 ms (Fig. 1a, left). This version included an implicit interval production component (750 ms) but was not explicitly designed to assess temporal perception. Hence, we complemented this approach with a “peak-interval” design, where the levers became functional only 30 s after the last reward was delivered. That is, only the first coordinated lever press was rewarded after 30 s. Following the reward delivery, the time counter reset, and the levers remained inactive for an additional 30 seconds (Fig. 1a, right). Our results indicate that during the first training sessions, the animals continuously lever-pressed during most of the 30 s period (Fig. 1b, left), and their overshoots lasted hundreds of milliseconds (Fig. 1c, d up), indicating poor temporal processing for both the perception of the 30-second-long interval and the production of the 750 ms coordinated movement. However, after training, animals limited their presses to a few seconds after the 30 s interval (Fig. 1b, right), indicating a good perception of the long interval. Conversely, lever presses were more accurate, with only a few milliseconds of overshoot (Fig. 1c, d bottom), indicating that the movement duration was adjusted to meet the experimental demands. We were also able to assess the coordination level (Fig. 1e) and the speed at which the left and right forelimbs moved (Fig. 1f). Then, to create learning curves, the movement-related variables (Fig. 1g–j) and the area under the curve (AUC) extracted from lever-pressing distributions (Fig. 1k) were formally quantified on a session-by-session basis, revealing progressive improvements as training evolved. Hence, with this approach, we were able to simultaneously assess movement parameters as well as two levels of temporal processing: the perception of elapsed time on the scale of dozens of seconds (peak interval) and the interval production on the scale of hundreds of milliseconds, enhancing our understanding of potential effects on movement, temporal processing, or both. Then, we explored the effects of systemic administrations of different doses of CP55940. Pharmacological sessions started after at least 30 training sessions. As reported previously [21], a dose-dependent effect was observed in movement duration and speed, with higher doses inducing slower and longer movements (Fig. 1l, m; Overshoot K-W, *df* = *4; X*^*2*^ = *36.27, p* < *0.0001*). Nonetheless, other execution parameters, such as interlimb correlation or movement onset variability, were slightly changed with the highest dose (Fig. 1n, o; interlimb correlation K-W, *df* = *4; X*^*2*^ = *18.98, p* = *0.0008;* BMOV K-W, *df* = *4; X*^*2*^ = *8.31, p* = *0.0809*). Longer movements could be interpreted as an underestimation of the 750 ms produced interval; however, when analyzing the behavior in the longer 30 s range, we observed a phenomenon that could be interpreted as an overestimation of the perceived interval. Higher doses of CP55940 resulted in lever presses homogeneously distributed throughout the entire interval (Fig. 1p), reflecting significantly higher values of the AUC (Fig. 1q; K-W, *df* = *4; X*^*2*^ = *42, p* < *0.0001*) and with cumulative peaks closer to or even under 30 s (Fig. 1r; K-W, *df* = *4; X*^*2*^ = *19.2, p* = *0.0005*). Given that our behavioral sessions lasted 40–60 min following cannabinoid injections, we considered the possibility of time-dependent effects due to drug kinetics or changes in reward expectation, as animals became progressively satiated. To address this, we re-analyzed our experimental sessions by comparing first and second halves. First, we calculated the average inter-trial interval and observed that it remained stable at approximately 30 s throughout sessions, with no significant differences between early and late trials (Fig. S1a). Similarly, both the peak interval (Fig. S1b) and the AUC (Fig. S1c) maintained consistent dose-response patterns across session halves without significant temporal differences. On the other hand, we observed an increase in overshoot during the second half across all conditions (including controls), but the dose-response relationship remained intact (Fig. S1d). These observations ruled out non-specific time-dependent effects of cannabinoid administration during behavioral sessions.

**Fig. 2:**
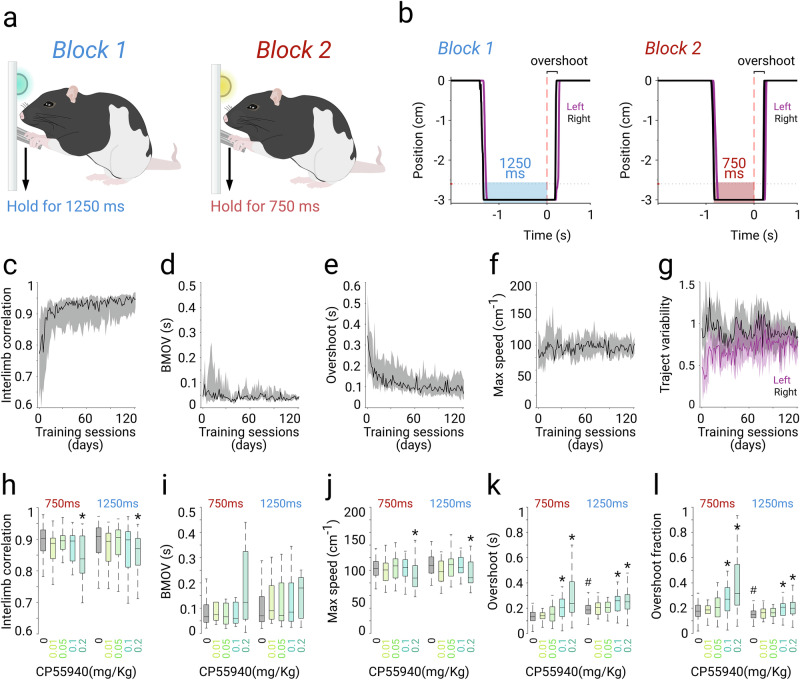
CP55940 effects on the production of two temporal intervals. **a** Schematic representation of the behavioral set-up. **b** Representative average trajectories (shaded area, 25^th^ and 75^th^ percentiles) of the left and right levers (color coded) aligned to the reward onset (dotted red line) during long (1250 ms) and short (750 ms) movement blocks. Average learning curves for a group of animals (*n* = 7) for the following variables: interlimb correlation (**c**), bilateral movement onset variability (BMOV, **d**), overshoot (**e**), maximum lever speed (**f**), and trajectory variability (**g**). Data for learning curves are presented as median (solid line) + 75th and 25th percentiles (shaded area). Box plot comparison of the effect of different doses of CP55940 (color coded) on the different bilateral execution variables for the two types of trials: 750 ms and 1250 ms (**h–l**). Boxplots represent median and 25^th^ and 75^th^ percentiles. * and # represent significant differences (LSD post hoc test, *p* < 0.05) against control (0 mg/kg) and 0.2 mg/kg conditions, respectively.

The previous data suggest that CP55940 may differentially affect temporal processing at the perception level (30 s range) and production level (750 ms range). However, they could also indicate that temporal production is not impaired, as movement parameters may covary (i.e., slow movements could seem longer). To address this possibility, we trained a new group of animals in the same original bimanual coordination protocol but introduced another modification. In this case, we did not use the peak-interval component but only manipulated the temporal production component in the range of hundreds of milliseconds. Here, instead of using only a holding time threshold of 750 ms, we also introduced a 1250 ms threshold. The two intervals were presented in 20 alternating trial blocks, and sessions always started with the longest threshold (Fig. 2a, b). We focused our analysis on the first 100 trials of the sessions, exhibiting consistent engagement in the task. After this, animals spontaneously took breaks and engaged in bouts of irregular numbers of trials until the end of the session. Learning curves in this version indicated that the animals progressively improved movement execution parameters, as attested by an increase in interlimb correlation (Fig. 2c), a decrease in BMOV (Fig. 2d) and overshoots (Fig. 2e). Movement speed (Fig. 2f) and individual forelimb variability (Fig. 2g) remained stable throughout the learning curve. We also assessed efficiency parameters, such as the amount of time to reach the first 100 trials in each session (Fig. S2a), the total number of trials per session (Fig. S2b), the effort to obtain a reward (Fig. S2c), and the number of attempts per reward (Fig. S2d). We observed that while the amount of time to reach the first 100 trials decreased with training, the other variables remained stable. Altogether, the learning curves indicated that, animals refined their movement-related parameters while maintaining stable engagement and efficiency to obtain rewards. After training in this version, animals produced movement trajectories with similar execution parameters (Fig. 2b–g) and overshoots in both types of trials (Fig. 2b, e). Similar overshoots indicate that the animals represented and quickly adapted to the duration of the movement from a long (1250 ms) to a short (750 ms) reach, as opposed to simply reacting to reward delivery or cues (i.e., reaction time). In this way, if animals control the interval production, movement duration must be close to the demanded interval, and the result would be better reflected in potential cannabinergic effects. In other words, if CP55950 induces an underestimation of interval production, it should impact both intervals proportionally. Conversely, if the slowness of movement were to impact the duration of movement, we could anticipate differential effects, such as longer movements for the 750 ms interval but not for the 1250 ms interval. After the training period ( ~ 150 sessions), we carried out pharmacological manipulations. Cannabinergic administrations did not significantly alter efficiency parameters, including the amount of time to reach the first 100 trials (Fig. S2e; K-W, *df* = *9; X*^*2*^ = *7.57, p* < *0.1089*), the total number of trials per session (Fig. S2f; K-W, *df* = *9; X*^*2*^ = *7.05, p* < *0.1332*), effort (Fig. S2g; K-W, *df* = *9; X*^*2*^ = *276.45, p* < *0.001;*), or the number of attempts to obtain a single reward (Fig. S2h; K-W, *df* = *9; X*^*2*^ = *4.48, p* = *0.877*; S-R-H*, Interval, H* = *76.85, p* < *0.001; Dose, H 2.49, p* = *0.6450*). However, consistent with previous observations [21, 27, 30], we found that systemic administrations of CP55940 produced dose-dependent motor impairments reflected in a decrease in interlimb correlation (Fig. 2h; K-W, *df* = *9; X*^*2*^ = *20.81, p* = *0.0135;* S-R-H*, Interval, H* = *2.05, p* = *0.1516; Dose, H 18.54, p* < *0.001*), without clear effects on bilateral movement onset variability (Fig. 2i; K-W, *df* = *9; X*^*2*^ = *10.15, p* = *0.3381;* S-R-H*, Interval, H* = *3.05, p* = *0.08; Dose, H* = *6.48, p* = *0.165*) but with a significant reduction of the maximum movement speed (Fig. 2j; K-W, *df* = *9; X*^*2*^ = *17.87, p* = *0.0367;* S-R-H*, Interval, H* = *3.584, p* = *0.058; Dose, H* = *12.85, p* = *0.012*). We also found a dose-dependent increase in overshoot in both intervals (Fig. 2k; K-W, *df* = *9; X*^*2*^ = *108.6, p* < *0.0001;* S-R-H*, Interval, H* = *61.19, p* < *0.001; Dose, H* = *42.94, p* < *0.001*); however, this increase appeared to be more robust in the production of the 750 ms interval. Since overshoot directly reflects movement duration, this observation indicates that the impact on movement duration is not proportional to the required interval. This was confirmed when we analyzed the overshoot fraction (Fig. 2l; K-W, *df* = *9; X*^*2*^ = *60.56, p* < *0.0001;* S-R-H*, Interval, H* = *19.55, p* < *0.001; Dose, H* = *39.54, p* < *0.001*). These results indicate that systemic administrations of CP55940 induced differential effects based on the interval, which is more consistent with impairments in speed control than with the production of temporal intervals. However, cannabinergic effects may be influenced by differential behavioral engagement or drug kinetics throughout the session. In this protocol, animals completed the first and last 100 trials in approximately the first 12 min or the last 32 min of each session, respectively (Fig. S2a, e; Fig. S3a). Therefore, we performed the same analyses focusing only on the trials of the 1250 ms condition, comparing the first 100 trials with the last 100 trials of each session. Interlimb correlation (Fig. S3b) or maximum speed (Fig. S3c), remained constant independently of the moment of the session, further confirming the stability of cannabinergic effects throughout the session. However, during late trials, overshoot and overshoot fraction did not exhibit significant differences between the control and cannabinergic conditions (Fig. S3d, e). Altogether, these results further support that cannabinergic effects are more closely related to the control of movement speed than to the production of specific temporal intervals. Finally, the two-interval alternating block schedule may induce an “averaged” overshoot between 750 and 1250 ms conditions, potentially compromising our interpretations. If this were the case, it would likely manifest as similar overshoot and overshoot fraction values in trials occurring near transitions between blocks. To evaluate this possibility, we conducted a within-block analysis, comparing the first 10 trials with the last 10 trials of each block. The data indicate no significant differences between these trial groups (Fig. S4a, b). Furthermore, the same pattern of control versus cannabinergic dosage was maintained in both early and late trials within each block (Figure S4a, b). These results suggest that the animals recognized the 750 ms or 1250 ms contexts and adapted their motor plan accordingly.

### Rats dynamically adjust their speed to maintain the spatiotemporal structure of a well-trained complex sequence of movements

**Fig. 3:**
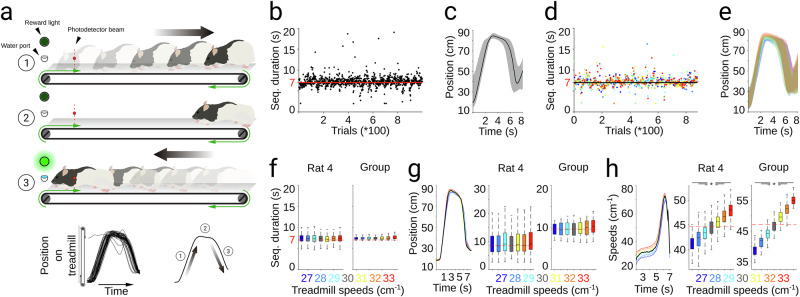
Spatiotemporal behavioral protocol. **a** Schematic representation of the different phases of the front-back-front strategy expressed by the animals in the spatiotemporal task (top). Trajectories for every trial and the average trajectory of a representative session are presented at the bottom. The different phases of the sequence are indicated in the average trajectory. Sequence duration (**b**, **d**) and representative average trajectories (**c**, **e**; shaded area represents + 75^th^ and 25^th^ percentiles) for a highly trained animal during behavioral execution in the basic single-speed (**b**, **c**) and multiple-speed phases (color coded) of training. **f** Sequence durations by groups of trials for a representative animal (left) and the group of animals (right) at different treadmill speeds (color-coded dots and boxes). **g** Color-coded representative average position trajectories (left) and trajectory differences (center, right) with respect to the stereotypical position trajectory at the central speed for a representative animal (center) and for the group of animals (right). **h** Same as in (**g**) but for speed. The central line and box in boxplots represent the median and 25^th^ and 75^th^ percentiles. Whiskers extend to the most extreme data points, excluding outliers. Bonferroni *post hoc* test ***p < 0.05.

**Fig. 4:**
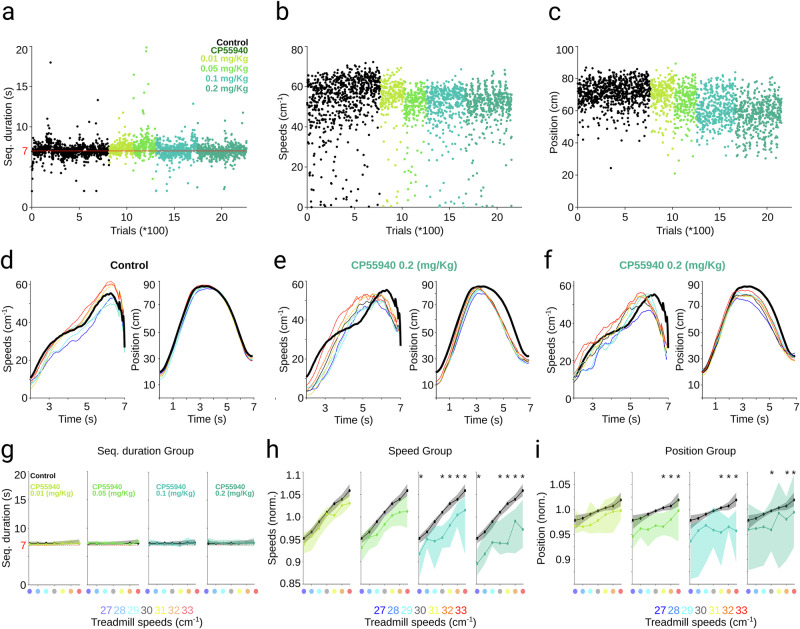
Effects of systemic administration of CP55940 on the execution of a timed motor sequence. Representative trial-by-trial sequence duration (dots represent individual trials; **a**), peak speed (**b**), and difference in position (**c**) for an animal under control conditions and different doses of CP55940 (color coded). **d–f** Representative average speed (left) and position trajectories (right) at different treadmill speeds (color coded) during trials under control (**d**) and the highest dose (**e**, **f**) of CP55940. **g–i** Group effects of the different doses of CP55940 (color coded) for sequence duration (**g**), speed (**h**), and position (**i**). Data are presented as median (solid line and dots) +75^th^ and 25^th^ percentiles (shaded area).

To further confirm our prior findings, we tested CP55940 effects in a new animal cohort trained to produce a complex motor sequence requiring precise timing and active speed control. We first trained rats to perform a stereotyped and 7-second timed sequence of locomotion decelerations and accelerations while running on a motorized treadmill [31, 32] (see methods). Animals developed a stereotypical strategy consisting of the following steps (Fig. 3a): 1) Animals started each trial at the front of the treadmill and learned to be passively transported to the rear part of the apparatus by the movement of the treadmill belt; 2) then, animals held their position in the back of the treadmill by trotting for a few seconds at the speed of the treadmill’s belt; 3) finally, animals performed a controlled acceleration across the treadmill to reach the front part again. This “front-back-front” strategy was extracted and analyzed from spatiotemporal trajectories reconstructed from high-speed video recordings on a trial-by-trial basis (Fig. 3a, bottom). Highly trained animals displayed low variability in sequence duration around goal time (Fig. 3b) and significantly stereotyped execution trajectories (Fig. 3c). Then, to understand how speed control or temporal representations interact and contribute to the general architecture of movement sequences, we implemented a second phase of training (at least 30 sessions). In each session, the treadmill speed (but not the temporal rule) varied randomly in a range of 27 to 33 cm/s on a trial-to-trial basis, so the animals were forced to dynamically adapt their locomotion speed based on their perception of the treadmill’s speed and on their representation of the 7 s temporal interval. We found that, independently of the treadmill speed, the rats accurately maintained the sequence duration around goal time (Fig. 3d) and displayed similar spatiotemporal trajectories (Fig. 3e). These effects were observed in representative sessions from individual animals (Fig. 3b–e) and the group (Fig. 3f, g; Rat 04, Time K-W, *df* = *6, X*^*2*^ = *16.71, p* = *0.01;* Position K-W, *df* = *6; X*^*2*^ = 14.7. Group, Time K-W, *df* = *6; X*^*2*^ = *4.1; p* = *0.663;* Position K-W, *df* = *6; X*^*2*^ = *3.67; p* = *0.721*). These data indicate that the animals adjusted their behavior to maintain the sequence duration, though the specific movement parameter involved remains unclear. For example, animals may have varied how long they stayed at the back of the treadmill based on its speed, or adjusted their speed during the final phase, accelerating in faster trials or decelerating in slower ones. To evaluate these possibilities, we calculated the speed peaks during the last acceleration across the treadmill. We found a linear relationship between the treadmill speeds and the peak speed reached by the animals, with a lower peak speed when the treadmill moved at lower speeds (27–29 cm/s) and a higher peak speed when the treadmill moved at higher speeds (31–33 cm/s). This behavior was observed in individual animals (Fig. 3h, center) and the group (Fig. 3h, right; Rat 04 K-W, *df* = *6; X*^*2*^ = *1683.2; p* < *0.001*. Group K-W, *df* = *6; X*^*2*^ = *226.22; p* < *0.001*). These data indicate that the animals adjusted their speed to preserve the sequence’s spatiotemporal structure, reflecting a preserved representation of the 7 s interval. Hence, this task may allow us to distinguish cannabinoid effects on speed control from on time representation. We conducted the same CP55940 pharmacological manipulations as in the two previous protocols. Contrary to our observations for the bimanual movement, we found that cannabinergic administrations did not induce significant changes in the duration of the movement sequence (Fig. 4a). Then, we analyzed the locomotion speed, finding a clear dose-dependent effect, with gradually slower accelerations associated with higher doses of CP55940 (Fig. 4b). This finding confirms that the mechanisms controlling behavioral timing and speed are different and can be differentially affected by cannabinoids. During the execution of the sequence, the temporal component can be linked to the animals’ speed, raising the question as to how the temporal component is unaffected if the animals are running slowly. This was clarified when analyzing the spatial component of the motor sequence. Here, we also observed a clear dose-dependent effect (Fig. 4c). In the control condition, spatiotemporal trajectories at different treadmill speeds overlapped (Fig. 4d, right), but speed trajectories showed a gradient, with higher and lower locomotion peaks corresponding to higher and lower treadmill speeds, respectively (Fig. 4d, left). However, with the higher doses of CP55940, the animals tended to express one of two strategies to compensate their slowness. Either they started the last acceleration almost as soon as they reached the rear end of the treadmill (Fig. 4e, right)—that is, they did not hold their position for as long as they did in the control condition— or they did not let themselves be transported to the far end of the apparatus (Fig. 4f, right). Both strategies combined with movement slowness, resulted in the animals completing the sequences close to the 7 s rule. As in the previous protocols, these effects were clearly visible in representative sessions from individual animals (Fig. 4a–f) and the group (Fig. 4g–i). These results suggest that, to maintain the target duration of the motor sequence under circumstances where cannabinoids cause a decrease in peak speeds, the animals implemented compensatory adjustments to their timing (not waiting for too long on the back of the treadmill) or their position on the treadmill (avoiding being carried entirely to the back). Altogether, this set of data confirms the existence of independent representations for space, speed control, and motor timing, and indicates that cannabinoids differentially affect these variables.

## Discussion

Our results showed that cannabinoid administration impaired the perception of long temporal intervals in the range of dozens of seconds while maintaining interval production in the range of hundreds of milliseconds (Figs. 1, 2). On the other hand, the same administrations systematically affected speed control independently of the spatiotemporal context (Figs. 3, 4), which is consistent with previous reports [17]. Previous studies have suggested that cannabinoids alter the perception of elapsed time, often leading to overestimations in tasks requiring interval discrimination [5–7, 9]. In agreement with this, we found that CP55940 systemic administrations led to an overestimation of elapsed time in a fixed-interval schedule task where rats were required to estimate a 30-second interval. This suggests that cannabinoids disrupt sensory-based time estimation, possibly due to their effects on cortico-striatal or cortico-hippocampal circuits, both of which are rich in CB1 receptor expression [16, 33] and have been implicated in interval timing [34–38]. On the other hand, in the two-interval production task, cannabinoid administration caused longer forelimb movements in the production of time intervals in the range of hundreds of milliseconds. This result is typically interpreted as an underestimation of the required time interval, implying that cannabinoids may also affect motor timing. However, when we used two production intervals (Fig. 2), the temporal effects were not proportional to the interval duration, suggesting a potential motor confound. To explore this possibility, we evaluated the effects of cannabinoids on a spatiotemporal motor task that required executing a well-learned sequence of movements with a tight temporal constraint of 7 s (goal time) (Fig. 3). Under these conditions, CP55940 injections produced no significant disruption in motor timing precision. More importantly, despite a clear dose-dependent reduction in locomotion speed, the rats compensated by adjusting their spatial trajectories, thereby ensuring that the total sequence duration remained close to the goal time (Fig. 4). These results not only confirm that the animals maintained a functional representation of the temporal interval, but also that they were able to sense the behavioral disparities induced by cannabinoids and compensate them by manipulating another execution variable, specifically position. Previous reports indicate that in this task, animals entrain their behavior to stable environmental variables, such as treadmill speed or length [18, 32, 39], which makes it difficult to adapt to unpredictable changes in these variables and raises questions about the explicit representation of the sequence duration. However, in our experiments, animals successfully adapted to subtle changes in treadmill speed. It is possible that this discrepancy arises from differences in task structure and training history. For example, in our training schedule, animals were first overtrained in a fixed-speed condition before transitioning to a random-speed protocol, which may have facilitated compensatory adjustments. Safaie et al. [39] observed impairments in speed adaptation when animals underwent training with extreme speed variations (2–40 cm/s). In this study, we utilized a treadmill speed range of 27–33 cm/s, which may facilitate more precise adaptations. Another possibility is that, in the spatiotemporal task, the time interval to estimate is implicitly embedded within a complex motor sequence that also includes rhythmic sensory feedback, such as the somatosensory input from the animal’s paws while running [31]. In contrast, during the two-interval production task, the sensory feedback that the animals may receive to guide their behavior by keeping the levers pressed with their forepaws is continuous.

What would be the neurobiological mechanism underlying these cannabinergic effects?

First, it is important to consider that the substantial differences in spatiotemporal ranges between our tasks (hundreds of milliseconds vs. dozens of seconds, or tens vs. hundreds of centimeters) may indicate the involvement of distinct neuronal mechanisms in temporal perception and production across both protocols. Nevertheless, prior evidence from our group and others suggests that the basal ganglia may be similarly involved in certain aspects of execution in both protocols. For instance, we have demonstrated that the dorsolateral striatum is essential for appropriate learning and expert execution in both treadmill-based and bilateral coordination-based protocols [21, 22, 31, 32]. Similarly, the spiking activity of individual neurons in the dorsolateral striatum is robustly correlated with movement speed and the animal’s position in both protocols [22, 32].On the other hand, striatal population activity has been proposed as a primary neural substrate for representing elapsed time across durations ranging from hundreds of milliseconds to dozens of seconds [13, 31, 34–36, 40]. Furthermore, manipulating these striatal dynamics—whether in the hundreds of milliseconds [13] or dozens of seconds range [31]—has been shown to induce behavioral changes consistent with the production of temporal intervals. This body of evidence suggests that similar neural mechanisms and structures may underlie the perception and production of temporal intervals in both protocols. On the other hand, previous reports suggest that a combination of molecular and structural locations implicating the basal ganglia, hippocampus, and cerebellum may be responsible for these effects. First, the robust slowness of movements observed in all our behavioral protocols is most likely related to the activation of CBr1, located in the terminals of the direct pathway neurons of the substantia nigra pars reticulata [21, 27, 41] resulting in unbalanced direct/indirect pathway activity, thereby enhancing inhibition of the motor thalamus [21, 42, 43]. Second, the effects on sensory timing may be related to CBr1 located in the hippocampus, basal ganglia, and/or cerebellum, since these regions have been strongly implicated in temporal processing [11, 13, 15, 31, 34, 38, 40, 44, 45] and exhibit the highest expression of CBr1 receptors in the brain [16, 33, 46]. While striatal and hippocampal circuits may be primarily involved in interval perception and production [38, 47, 48], cerebellar circuits could contribute to compensatory mechanisms, allowing animals to adjust movement execution despite cannabinoid-induced slowness of movements [45, 49, 50]. The overestimation of long intervals in the fixed-interval task supports a role for cannabinoids in disrupting sensory timing. However, timing in well-learned motor sequences remains preserved, likely due to compensatory strategies that adjust spatial parameters to maintain temporal precision. Furthermore, interval production effects are most likely related to nonspecific effects related to slowness in motor execution. Taken together, our results suggest that cannabinoids selectively impair time perception but not production of temporal intervals, and their effects depend on the specific timescale and task demands. Future studies should examine the specific neural circuits underlying these differential effects.

## Supplementary information

Supplemental material

## Data Availability

The data supporting the main findings of this study are available at: 10.5281/zenodo.16755556. Raw datasets are available from the corresponding author upon reasonable request.
