# Supplementary material for: Differential cannabinergic effects on temporal perception and production
Source: Neuropsychopharmacology. 2025 Oct 13;51(3):672–81. doi: 10.1038/s41386-025-02262-5 (PMC12824324; doi:10.1038/s41386-025-02262-5)
Supplement: Supplementary file 1 — Supplemental material [file 41386_2025_2262_MOESM1_ESM.pdf]

Differential cannabinergic effects on temporal perception and production

Mario G. Martínez-Montalvo, Diana I. Ortega-Romero, Ana S. Báez-Cordero, Oswaldo Sánchez-Lobato, Claudia I. Perez, Pavel E. Rueda-Orozco

Supplementary Figures

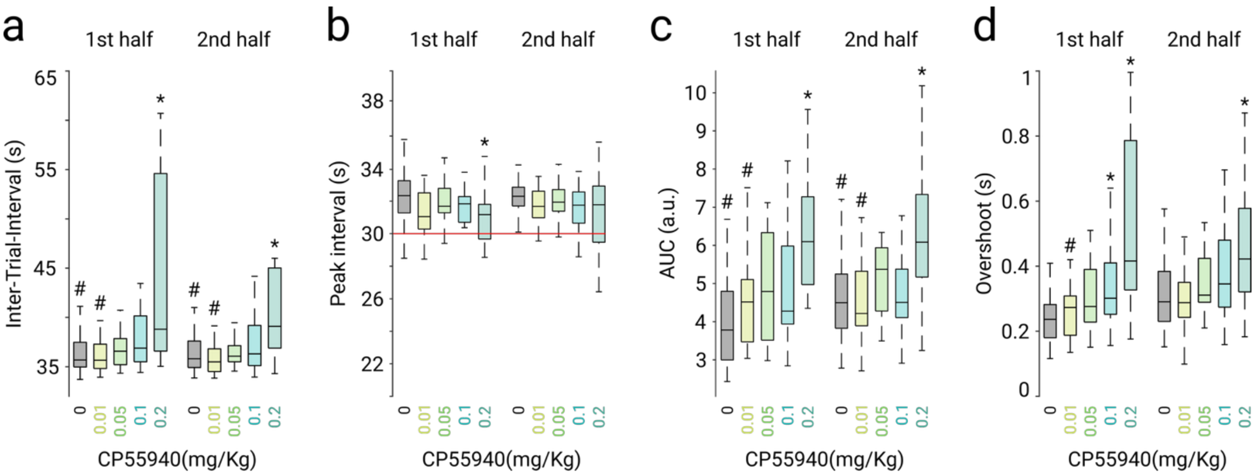

Figure S1. CP55940 effects on the perception and production of temporal intervals on early and late trials. Boxplot comparison of the effect of the CP55940 doses (color coded) on inter trial intervals (**a**), moment of the peak lever response around reward onset (**b**), peri-reward histogram area under the curve (**c**) and (**r**, peak interval). Boxplots represent median and 25th and 75th percentiles. Boxplots represent median and 25th and 75th percentiles. * and # represent significant differences (LSD post hoc test, $p < 0.05$) against control (0 mg/kg) and 0.2 mg/kg conditions, respectively.

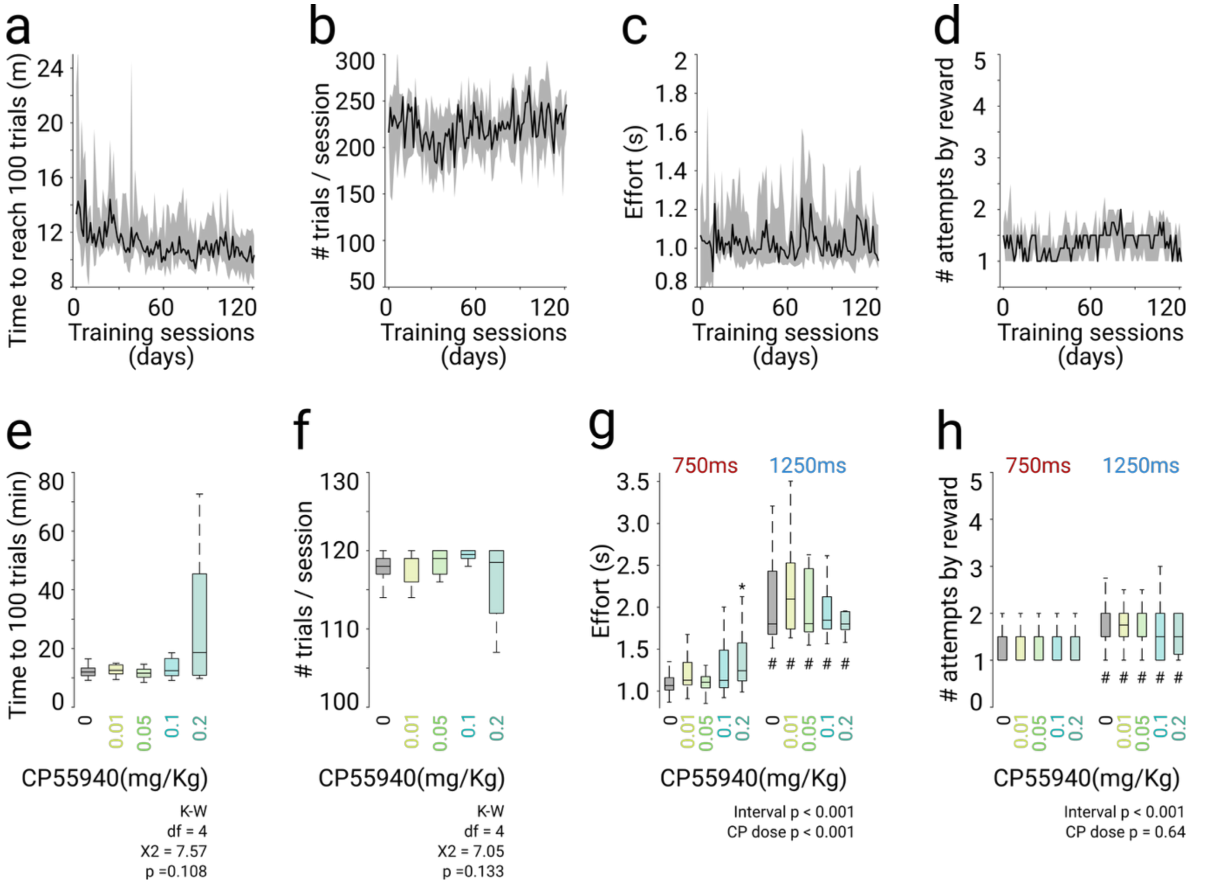

Figure S2. CP55940 effects on the efficiency variables in the two-intervals protocol. Average learning curves for a group of animals (n = 7) for the following variables: time to reach the first 100 trials (**a**), total number of trials per session (**b**), effort (**c**), and number of attempts by reward (**d**) Data for learning curves are presented as median (solid line) + 75th and 25th percentiles (shaded area). Box plot comparison of the effect of different doses of CP55940 (color coded) on the different efficiency variables (**e-h**). Boxplots represent median and 25th and 75th percentiles. * represents significant differences (LSD post hoc test, p < 0.05) against control within the same condition. # represents significant differences (LSD post hoc test, p < 0.05) of the same dose between conditions (750 ms vs. 1250 ms). K-W values are indicated in panels e, f and Scheirer-Ray-Hare test p values are indicated in panels g, h.

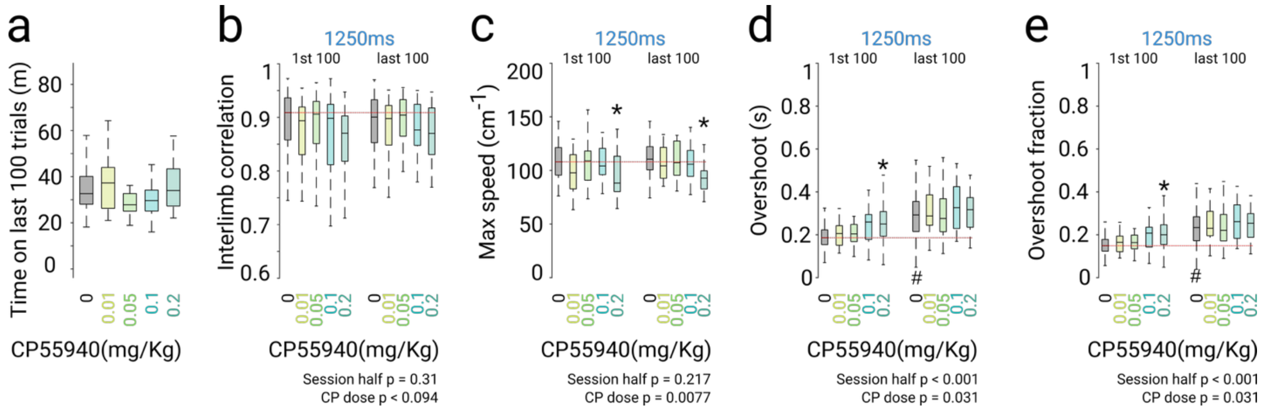

Figure S3. CP55940 effects on the production of two temporal intervals during early and late trials. Box plot comparison of the effect of different doses of CP55940 (color coded) on the time to perform the last 100 trials of the session (**a**), interlimb correlation (**b**), maximum lever speed (**c**), overshoot (**d**), and overshoot as fraction of the demanded interval (**e**). Boxplots represent median and 25th and 75th percentiles. * and # represent significant differences (LSD post hoc test, $p < 0.05$) against control within the same condition (0 mg/kg) or between conditions, respectively. Scheirer-Ray-Hare test p values are indicated under the corresponding panel.

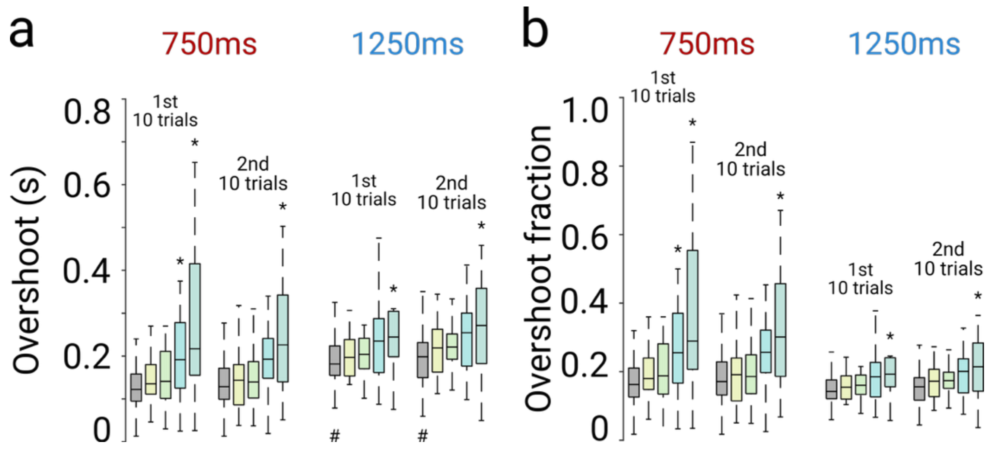

Figure S4. CP55940 effects on overshoots within blocks of trials. Box plot comparison of the effect of different doses of CP55940 (color coded) on overshoot (a) and overshoot as fraction of the demanded interval (**b**) divided groped as the first and las last 10 trials of each block on each 750 and 1250 ms conditions. Boxplots represent median and 25th and 75th percentiles. * and # represent significant differences (LSD post hoc test, $p < 0.05$) against control within the same condition (0 mg/kg) or between conditions, respectively.
